# A tale of two targets: examining the differential effects of posterior cingulate cortex- and amygdala-targeted fMRI-neurofeedback in a PTSD pilot study

**DOI:** 10.3389/fnins.2023.1229729

**Published:** 2023-11-29

**Authors:** Jonathan M. Lieberman, Daniela Rabellino, Maria Densmore, Paul A. Frewen, David Steyrl, Frank Scharnowski, Jean Théberge, Niki Hosseini-Kamkar, Richard W. J. Neufeld, Rakesh Jetly, Benicio N. Frey, Tomas Ros, Ruth A. Lanius, Andrew A. Nicholson

**Affiliations:** ^1^Atlas Institute for Veterans and Families, Ottawa, ON, Canada; ^2^Department of Psychiatry and Behavioural Neurosciences, McMaster University, Hamilton, ON, Canada; ^3^Imaging, Lawson Health Research Institute, London, ON, Canada; ^4^Department of Neuroscience, Western University, London, ON, Canada; ^5^Department of Psychiatry, Western University, London, ON, Canada; ^6^Department of Psychology, Western University, London, ON, Canada; ^7^Department of Cognition, Emotion, and Methods in Psychology, University of Vienna, Vienna, Austria; ^8^Department of Medical Biophysics, Western University, London, ON, Canada; ^9^Department of Diagnostic Imaging, St. Joseph’s Healthcare, London, ON, Canada; ^10^The University of Ottawa Institute of Mental Health Research, Royal Ottawa Hospital, University of Ottawa, Ottawa, ON, Canada; ^11^Department of Psychology, University of British Columbia, Kelowna, BC, Canada; ^12^Mood Disorders Treatment and Research Clinic, St. Joseph’s Healthcare Hamilton, Ontario, ON, Canada; ^13^Department of Neuroscience and Psychiatry, University of Geneva, Geneva, Switzerland; ^14^School of Psychology, University of Ottawa, Ottawa, ON, Canada

**Keywords:** post-traumatic stress disorder, fMRI neurofeedback, posterior cingulate cortex, amygdala, default mode network

## Abstract

**Introduction:**

Real-time fMRI-based neurofeedback (rt-fMRI-NFB) is a non-invasive technology that enables individuals to self-regulate brain activity linked to neuropsychiatric symptoms, including those associated with post-traumatic stress disorder (PTSD). Selecting the target brain region for neurofeedback-mediated regulation is primarily informed by the neurobiological characteristics of the participant population. There is a strong link between PTSD symptoms and multiple functional disruptions in the brain, including hyperactivity within both the amygdala and posterior cingulate cortex (PCC) during trauma-related processing. As such, previous rt-fMRI-NFB studies have focused on these two target regions when training individuals with PTSD to regulate neural activity. However, the differential effects of neurofeedback target selection on PTSD-related neural activity and clinical outcomes have not previously been investigated.

**Methods:**

Here, we compared whole-brain activation and changes in PTSD symptoms between PTSD participants (*n* = 28) that trained to downregulate activity within either the amygdala (*n* = 14) or the PCC (*n* = 14) while viewing personalized trauma words.

**Results:**

For the PCC as compared to the amygdala group, we observed decreased neural activity in several regions implicated in PTSD psychopathology – namely, the bilateral cuneus/precuneus/primary visual cortex, the left superior parietal lobule, the left occipital pole, and the right superior temporal gyrus/temporoparietal junction (TPJ) – during target region downregulation using rt-fMRI-NFB. Conversely, for the amygdala as compared to the PCC group, there were no unique (i.e., over and above that of the PCC group) decreases in neural activity. Importantly, amygdala downregulation was not associated with significantly improved PTSD symptoms, whereas PCC downregulation was associated with reduced reliving and distress symptoms over the course of this single training session. In this pilot analysis, we did not detect significant between-group differences in state PTSD symptoms during neurofeedback. As a critical control, the PCC and amygdala groups did not differ in their ability to downregulate activity within their respective target brain regions. This indicates that subsequent whole-brain neural activation results can be attributed to the effects of the neurofeedback target region selection in terms of neurophysiological function, rather than as a result of group differences in regulatory success.

**Conclusion:**

In this study, neurofeedback-mediated downregulation of the PCC was differentially associated with reduced state PTSD symptoms and simultaneous decreases in PTSD-associated brain activity during a single training session. This novel analysis may guide researchers in choosing a neurofeedback target region in future rt-fMRI-NFB studies and help to establish the clinical efficacy of specific neurofeedback targets for PTSD. A future multi-session clinical trial of rt-fMRI-NFB that directly compares between PCC and amygdala target regions is warranted.

## 1 Introduction

Over the past two decades, interest in real-time functional magnetic resonance imaging-based neurofeedback (rt-fMRI-NFB) has grown rapidly, largely owing to recent developments in real-time data processing and pattern analysis (Watanabe et al., 2017). rt-fMRI-NFB is a non-invasive brain-computer interface that enables individuals to self-regulate specific brain networks and functions, which can be particularly beneficial for treating various psychiatric conditions. Indeed, several studies have implemented rt-fMRI-NFB in a range of prevalent psychiatric conditions (Linden et al., 2012; Li et al., 2013; Schoenberg and David, 2014; Hamilton et al., 2016; Young et al., 2017; Mehler et al., 2018), including post-traumatic stress disorder (PTSD) (Gerin et al., 2016; Misaki et al., 2018, 2019, 2021; Nicholson et al., 2016a, 2018, 2021; Zotev et al., 2018; Zweerings et al., 2018, 2020; Chiba et al., 2019; Weaver et al., 2020; Lieberman et al., 2023; Zhao et al., 2023). In designing clinical rt-fMRI-NFB studies, multiple methodological factors require critical consideration; however, one of the most crucial decisions is the selection of the neurophysiological basis by which to generate the neurofeedback signal. While there are several possible approaches in this regard – including multi-voxel activation (i.e., decoded neurofeedback) and functional connectivity-based neurofeedback – the majority of previous rt-fMRI-NFB studies in PTSD have selected a target region as the basis for the neurofeedback signal (Gerin et al., 2016; Nicholson et al., 2016a, 2021; Misaki et al., 2018, 2019; Zotev et al., 2018; Zweerings et al., 2018; Weaver et al., 2020; Zhao et al., 2023).

In a clinical context, the selection of a neurofeedback target region is primarily informed by the neurobiological characteristics and desired clinical outcomes of the psychiatric condition being studied. Two main approaches are used for selecting a target region in the majority of clinical studies: the altered process approach and the compensatory process approach (Sulzer et al., 2013; Young et al., 2021). In the altered process approach, rt-fMRI-NFB is used to select regions with altered function or connectivity that are linked to the psychiatric condition being studied. This approach requires prior knowledge of the pathophysiological mechanisms underlying the condition to inform the choice of target region and the direction of regulation (i.e., upregulate vs. downregulate). The guiding assumption of this approach is that by normalizing pathophysiological alterations in brain activity, participants may be able to alleviate associated symptoms (Sulzer et al., 2013; Young et al., 2021). Alternatively, in the compensatory process approach, rt-fMRI-NFB is used to train participants to overcome impaired functionality by engaging compensatory neural mechanisms that have been well studied in healthy populations (Sulzer et al., 2013; Young et al., 2021). For instance, the putative brain regions underlying automatic and voluntary emotion regulation may serve as effective targets for neurofeedback training for several clinical populations in which emotion regulation deficits are present (Sulzer et al., 2013). Despite having these approaches available, it remains unclear how to select between several possible target regions that are all of neurobiological relevance to a particular psychiatric condition. Indeed, in the rt-fMRI-NFB literature, significant heterogeneity exists in the selection of target regions, even among studies with similar populations or clinical/behavioral objectives (Thibault et al., 2018). For instance, in a recent systematic review, multiple target regions (i.e., amygdala, insula, anterior cingulate cortex [ACC], prefrontal cortex [PFC]) were investigated across rt-fMRI-NFB studies in PTSD, depression, and anxiety-based disorders, all with the shared goal of improving emotion regulation capacities (Linhartová et al., 2019). This heterogeneity in neurofeedback target selection is unsurprising as psychiatric conditions, including PTSD, involve complex neurobiological mechanisms in which pathophysiological alterations extend across several distinct brain regions.

Extensive research has established a strong link between PTSD symptoms and multiple functional disruptions in the brain (e.g., Lanius et al., 2015; Tursich et al., 2015; Fenster et al., 2018). Indeed, both at rest and during trauma-related processing, hyperactivity within the amygdala – a brain region associated with emotion generation and processing – has been strongly associated with PTSD symptoms (Lanius et al., 2010, 2015; Etkin et al., 2011; Mickleborough et al., 2011; Patel et al., 2012; Birn et al., 2014; Yehuda et al., 2015; Aghajani et al., 2016; Koch et al., 2016; Fenster et al., 2018; Fitzgerald et al., 2018). There are also PTSD-associated alterations in functional connectivity between the amygdala and the cingulate cortex (including the ACC), insula, and PFC (Fonzo et al., 2010; Rabinak et al., 2011; Sripada et al., 2012; Birn et al., 2014; Brown et al., 2014; Nicholson et al., 2015, 2016b). In particular, negative medial PFC-amygdala connectivity is associated with PTSD symptom severity during trauma-related emotion processing (Stevens et al., 2013; Jin et al., 2014; Sadeh et al., 2014; Wolf and Herringa, 2016). Taken together, PTSD-associated emotion dysregulation appears to result from a hyperactive limbic system (i.e., amygdala, insula) driven by the loss of top-down inhibition from frontal brain regions (i.e., PFC, ACC) (Rauch et al., 2006; Lanius et al., 2010; Shin and Liberzon, 2010; Aupperle et al., 2012; Pitman et al., 2012; Admon et al., 2013; Ronzoni et al., 2016). Given this neurobiological account of PTSD, it is fitting that the majority of previous rt-fMRI-NFB studies in PTSD have targeted the amygdala, wherein participants were trained to either downregulate regional activity during negative emotion induction (Gerin et al., 2016; Nicholson et al., 2016a, 2018; Zhao et al., 2023) or upregulate regional activity during positive emotion induction (Misaki et al., 2018, 2019; Zotev et al., 2018). Other studies have taken a related approach in which individuals with PTSD were trained to upregulate frontal brain regions involved in emotion regulation (Zweerings et al., 2018, 2020). For instance, in one study, participants upregulated left lateral PFC activity while performing cognitive reappraisal to reduce their emotion response to negative scenes (Zweerings et al., 2020). However, it is increasingly apparent that the fronto-limbic model, as previously described, does not explain the full range of symptoms experienced by PTSD patients or account for alterations within other brain regions (e.g., Akiki et al., 2017; Fenster et al., 2018; Kamiya and Abe, 2020). Therefore, exploration of novel neurofeedback targets in PTSD is of critical importance.

The posterior cingulate cortex (PCC), the major hub of the posterior default mode network (DMN) (Greicius et al., 2003; Buckner et al., 2008; Spreng et al., 2009; Qin and Northoff, 2011; Brewer et al., 2013), is another brain region that is critically implicated in PTSD psychopathology. Among individuals with PTSD, there are alterations in functional connectivity of the PCC and the DMN both at rest (Akiki et al., 2017, 2018, Bluhm et al., 2009; Sripada et al., 2012; Lanius, 2015; Tursich et al., 2015; Yehuda et al., 2015; Koch et al., 2016; Hinojosa et al., 2019; Nicholson et al., 2020a) and during executive functioning tasks (Daniels et al., 2010; Melara et al., 2018). Importantly, hyperactivity within the PCC during the reliving and reexperiencing of trauma-related autobiographical memories in PTSD has also been reported (Ramage et al., 2013; Frewen et al., 2017; Awasthi et al., 2020; Thome et al., 2020), including in a recent meta-analysis (Thome et al., 2020). In a longitudinal study, PTSD patients with recent exposure to traumatic events (i.e., within the past 2 months) demonstrated greater activation in the right PCC as compared to control participants in response to trauma-related cues (Ke et al., 2016). Notably, at a two-year follow-up, decreased activation in the PCC during a trauma provocation task was also predictive of PTSD symptom improvement (Ke et al., 2016). Taken together, these findings highlight the PCC’s clinical relevance in PTSD psychopathology and suggest that regulating activity within the region via rt-fMRI-NFB may generate positive clinical outcomes. Critically, however, very few studies have examined the neurobiological mechanisms associated with the regulation of the PCC with rt-fMRI-NFB (Zhang et al., 2013; Garrison et al., 2013a,b; Kirlic et al., 2022; Yu et al., 2022), with only one study examining the regulation of this region in PTSD (Nicholson et al., 2021; Lieberman et al., 2023).

Previously, our group has used rt-fMRI-NFB to train individuals with PTSD to downregulate activity within both the amygdala and PCC during an identical trauma provocation paradigm in which individuals viewed personalized trauma words (Nicholson et al., 2016a, 2018, 2021; Lieberman et al., 2023). With regard to amygdala targeted neurofeedback, the involvement of the prefrontal cortex and its role in emotion regulation may be critical for facilitating successful downregulation. Indeed, our previous study showed that amygdala downregulation was associated with increased neural activity within the PFC (i.e., dlPFC, vlPFC), increased bidirectional amygdala-PFC (i.e., dlPFC, dmPFC) effective connectivity, and increased recruitment of the left central executive network (CEN; including the bilateral dlPFC) (Nicholson et al., 2016a, 2018). On the other hand, PCC downregulation was associated with simultaneous widespread decreases in neural activity within several brain regions implicated in PTSD psychopathology – namely, the dmPFC, postcentral gyrus, amygdala/hippocampus, cingulate cortex, and temporal pole/gyri (Nicholson et al., 2021). Additionally, PCC downregulation was also associated with increased PCC connectivity with the dmPFC, vmPFC, posterior insula, and amygdala (Lieberman et al., 2023). Hence, PCC downregulation may help to restore connectivity between functionally segregated posterior and anterior DMN structures and may also result in the concomitant regulation of brain regions involved in emotion generation/processing (i.e., amygdala) and embodiment (i.e., insula). Critically, however, no studies to date have conducted a direct comparison between multiple rt-fMRI-NFB target regions (i.e., the amygdala and the PCC) among individuals with PTSD. This novel analysis may help to elucidate unique neural mechanisms underlying rt-fMRI-NFB regulation and establish the clinical efficacy of specific neurofeedback targets.

### 1.1 Current study

In the current study, we aimed to explore differential neural mechanisms associated with rt-fMRI-NFB targeting the amygdala or PCC among individuals with PTSD. To do so, we compared whole-brain activation patterns between two groups of PTSD participants, who were trained to downregulate activity within either the amygdala or the PCC during an identical trauma provocation paradigm. Based on previous studies examining patterns of activation and connectivity associated with amygdala and PCC downregulation with rt-fMRI-NFB (Gerin et al., 2016; Nicholson et al., 2016a, 2018, 2021; Misaki et al., 2018, 2019; Zotev et al., 2018; Lieberman et al., 2023; Zhao et al., 2023), we expected to observe unique neural mechanisms involved in the downregulation of these two target regions when comparing them directly. More specifically, we hypothesized that amygdala downregulation would be associated with increased neural activity in emotion regulation areas within the prefrontal cortex, as compared to the PCC group. In contrast, we hypothesized that PCC downregulation would be associated with concomitant widespread decreases in neural activity within regions involved in PTSD psychopathology, as compared to the amygdala group.

## 2 Methods

### 2.1 Participants

The sample for this study consisted of *n =* 28 participants with PTSD (Table 1) who were trained to downregulate activity within one of two target regions – either the amygdala (*n* = 14) or the PCC (*n* = 14) – using rt-fMRI-NFB. While not analyzed here, there was also a group of healthy control participants who were trained to downregulate activity within the PCC (*n* = 15) (Nicholson et al., 2021). The sample size of this pilot investigation was based on study feasibility during the recruitment period. All participants were recruited via clinician referrals, community organizations, and posters throughout the London, Ontario community. Inclusion criteria included a current primary diagnosis of PTSD as measured by the Clinician-Administered PTSD Scale (CAPS-5) (Weathers et al., 2018) and the Structured Clinical Interview for DSM-5 (SCID) (First, 2015). Exclusion criteria included: ongoing or recent (within the previous three months) alcohol or substance use disorders, suicidal ideations, self-injurious behaviors requiring medical attention, lifetime diagnoses of bipolar or psychotic disorders, previous biofeedback treatment, noncompliance with 3 T fMRI safety guidelines, untreated medical conditions, pregnancy, previous head injury with loss of consciousness, and neurological or pervasive developmental disorders. All scanning took place at the Lawson Health Research Institute in London, Ontario. This research was approved by the Research Ethics Board at Western University, and all participants provided written informed consent. A CRED-nf checklist (Ros et al., 2020) summarizing experimental design was completed via the standardized CRED-nf online tool^1^ and is provided as Supplementary material.

**Table 1:** Demographic and clinical information.

Measure	PCC (*n* = 14)	Amygdala (*n* = 14)
Age	49.50 (±5.11)	48.08 (±9.78)
**Biological sex**	**6F, 8M**	**10F, 4M**
**CAPS-5**	**43.21 (±8.26)**	**31.70 (±9.42)**
BDI	32.14 (±12.55)	26.60 (±13.33)
CTQ	61.50 (±25.84)	60.64 (±16.58)
MDI	87.36 (±28.23)	68.20 (±27.12)
Psychotropic medication	10	11

Bolded measures (i.e., biological sex, CAPS-5) were found to differ significantly between groups and were included as covariates in subsequent analyses. Values in parentheses indicate standard deviation. CAPS-5, Clinician-Administered PTSD Scale (version 5); BDI, Beck’s Depression Inventory; CTQ, Childhood Trauma Questionnaire (*none or minimal childhood trauma* = 25–36, *moderate* = 56–68, extreme trauma > 72); MDI, Multiscale Dissociation Inventory.

Prior to scanning, participants completed several clinical assessments, including the Beck’s Depression Inventory (BDI) (Beck et al., 1997), the Childhood Trauma Questionnaire (CTQ) (Bernstein et al., 2003) and the Multiscale Dissociation Inventory (MDI) (Briere et al., 2005). After each of the fMRI neurofeedback runs, participants completed the Response to Script Driven Imagery Scale (RSDI) (Hopper et al., 2007a), which included the following symptom subscales: reliving, distress, physical reactions, dissociation, and emotional numbing.

There were no significant differences between the groups in terms of age, or scores on the BDI, CTQ, and MDI (Table 1). However, there were significant differences between the groups in terms of biological sex (amygdala group: 4 males, 10 females; PCC group: 8 males, 6 females) and CAPS-5 scores (amygdala group*: M* = 31.7, SD = 9.4; PCC group: *M* = 43.2, SD = 8.3) (Table 1). As such, both biological sex and CAPS-5 scores were included as covariates within subsequent analyses. Notably, there was a single missing data point for one participant’s CAPS-5 score. For the purposes of including CAPS-5 scores as a covariate in our analyses, we used the expectation–maximization (EM) algorithm to impute a value for this missing data point. To implement the EM algorithm, we used Python 3.9 and the scikit-learn library. Specifically, we used the ‘LinearRegression’ function to create a linear regression model, which we trained on the observed data from all participants (i.e., age, biological sex, CAPS-5 scores). We then used the trained model to impute a value for the missing data point for use as a covariate only. Among several possible approaches for imputing the missing data point, the EM algorithm with a linear regression model was optimal given the observed data. Previous publications by our group have analyzed the imaging data for each participant group separately (Nicholson et al., 2016a, 2018, 2021; Lieberman et al., 2023), but no previous study has assessed the differential neural and clinical effects associated with PCC as compared to amygdala-targeted fMRI-NFB.

### 2.2 Real-time fMRI neurofeedback protocol

**Figure 1:**
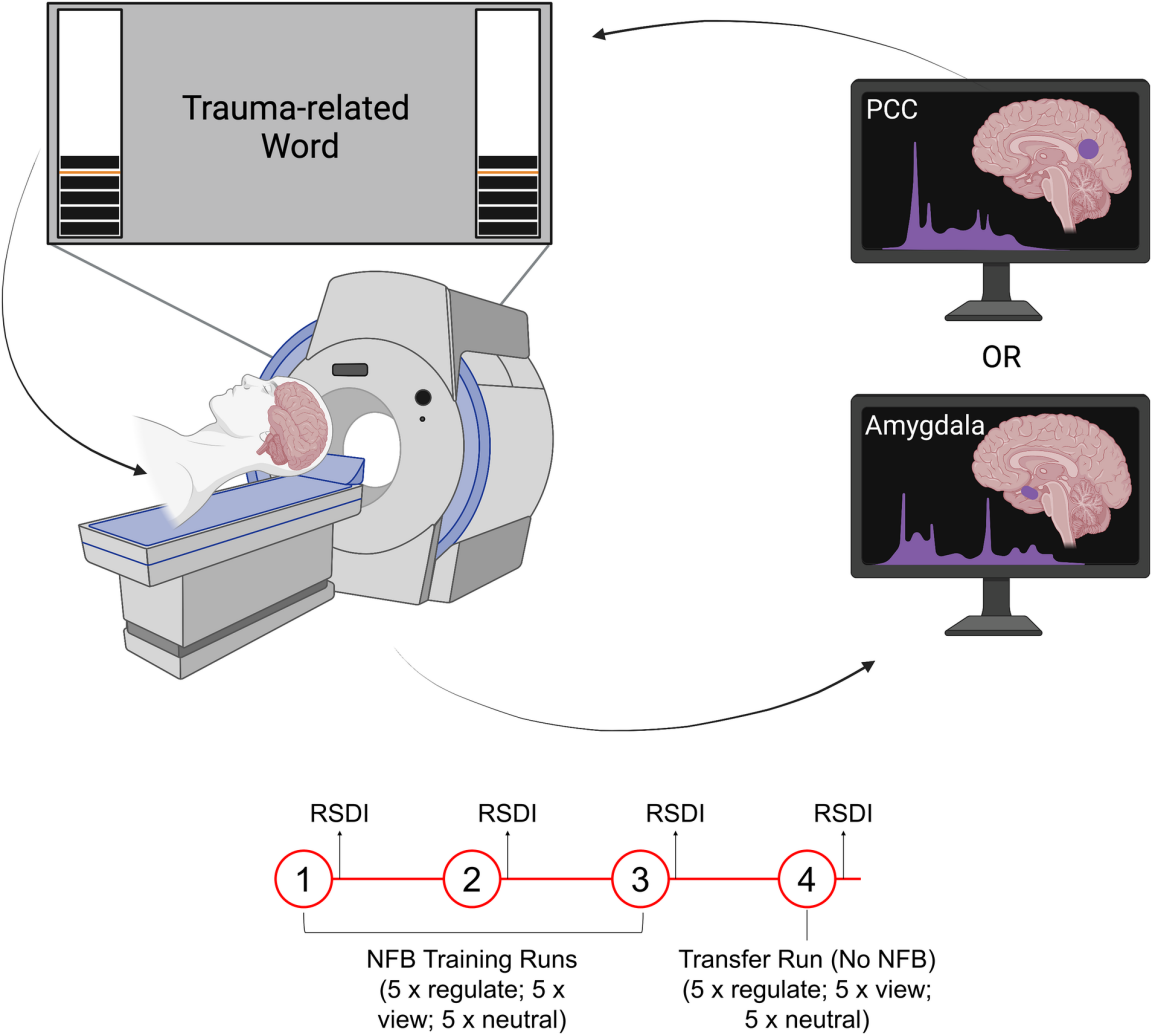
Illustration of the rt-fMRI-NFB experimental set-up. The neurofeedback signal took the form of a virtual thermometer whose level changed in response to fluctuating activity within the neurofeedback target region (PCC or amygdala). Participants viewed the neurofeedback signal during a trauma provocation paradigm while they were in the scanner. All participants completed three neurofeedback training runs, followed by a transfer run, in which they were not shown the neurofeedback signal. Figure adapted with permission from Nicholson et al. (2021).

All participants underwent an identical experimental protocol and neurofeedback paradigm, with the exception of the neurofeedback target region (i.e., the amygdala or PCC) (Figure 1). During neurofeedback training, participants were presented with a signal corresponding to BOLD activation within the neurofeedback target region. This neurofeedback signal was presented as a virtual thermometer on both sides of the MRI screen that was visible to participants while they were inside the scanner. The bars on the thermometer increased or decreased in correspondence to changes in BOLD activation within the neurofeedback target region. Participants were instructed that they would be regulating an area of the brain related to emotion. To avoid biasing the selection and usage of regulatory mental strategies by participants, no specific instructions were provided on how to regulate the neurofeedback target region (Paret et al., 2014, 2016a; Nicholson et al., 2016a, 2018, 2021). Rather, participants were instructed to use whichever strategies they personally found to work best for regulating the neurofeedback signal. Participants were asked to focus their gaze directly on the presented word for the duration of each condition and to use their peripheral vision to monitor the thermometers. Participants were also informed that the neurofeedback signal lags behind their brain activity by approximately 6–8 s (due to the BOLD signal time lag).

Our neurofeedback protocol consisted of three conditions: regulate, view, and neutral. In the *regulate* condition, participants were instructed to decrease the neurofeedback signal while viewing a personalized trauma-related word. In the *view* condition, participants viewed a trauma-related word but were instructed to respond naturally and not attempt to exert regulatory control over the neurofeedback signal. In the *neutral* condition, participants viewed a personalized neutral word and were instructed to respond naturally and not attempt to exert regulatory control over the neurofeedback signal. Personalized words were selected with the guidance of a trauma-informed clinician (*n* = 10 for both trauma-related and neutral words). Participants selected trauma-related words that were related to their individual experiences of trauma. The trauma-related words were matched on subjective units of distress to control for between subject/group variability. Stimuli were presented using Presentation software from Neurobehavioral Systems.

The experimental design included three consecutive neurofeedback training runs, followed by a single transfer run. In the transfer run, participants underwent an identical protocol except they were not shown a neurofeedback signal. Each run lasted 9 min and included 15 trials (5 per condition). The timing for all trials was as follows: 2 s for instructions, followed by 24 s for the condition, and then a 10 s implicit rest with an intertrial fixation cross. All trials were counterbalanced.

### 2.3 Real-time signal processing for neurofeedback

For all participants, we performed identical procedures to present real-time neural activation of the neurofeedback target region via a thermometer display. First, we imported anatomical scans into BrainVoyager (version QX2.4, Brain Innovations), skull-stripped and transformed them into Talairach space. We then added the normalization parameters into Turbo-BrainVoyager (TBV, version 3.0, Brain Innovations) which was the software used for real-time processing and analysis of BOLD signals. During real-time signal processing, TBV detected and corrected for small head movements (via rigid body transformation to the first recorded volume) and conducted spatial smoothing (4-mm full-width-half-maximum; FWHM). We discarded the first two volumes of the functional scans before real-time processing. Next, we defined the neurofeedback target region using TBV. For the amygdala, we used a bilateral anatomical mask from the PickAtlas software (WFU Pickatlas). For the PCC, we used a 6 mm sphere at the coordinate (MNI: 0–50 20) (Bluhm et al., 2009). In both cases, we then used the “best voxel selection” tool in TBV to calculate the BOLD signal amplitude in the defined target area. This method identifies the 33% most active (i.e., the highest beta-values) voxels for the *view* > *neutral* contrast. The first two trials of each neurofeedback run were the view and neutral conditions, which allowed us to select voxels based on the *view* > *neutral* contrast. This selection was dynamically updated throughout the duration of training. Indeed, as outlined in previous publications (Paret et al., 2014, 2016b; Nicholson et al., 2016a, 2018), dynamic voxel selection is based on (a) the voxel with the largest beta value, and (b) the magnitude of deviation from the mean of all condition betas (Goebel, 2014). This method ensures that there are no inter-subject differences in the number of voxels used for signal extraction. Additionally, it accounts for slight shifts in anatomical delineation resulting from changes in alignment across runs and/or movement-related slice shifts. The neurofeedback signal was calculated as the mean of the processed BOLD signal over the included voxels within the target brain region. In order to smooth out rapid BOLD signal fluctuations, the neurofeedback signal shown to participants via thermometer display was the mean of the neurofeedback signal of the current and 3 preceding TRs (Paret et al., 2014, 2016b; Nicholson et al., 2016a, 2021). At the start of each trial, the mean of the neurofeedback signal of the first 4 TRs (preceding stimuli onset) were utilized as a baseline and shown to participants as an orange line on the thermometer display. Subsequently, the level of the thermometer was continuously updated (at each TR) and shown to participants throughout the 3 neurofeedback training runs. Each segment of the thermometer corresponded to a 0.2% change in BOLD activation, with a maximum range of +2.8% and −1.2% from baseline (Paret et al., 2014, 2016b; Nicholson et al., 2016a, 2018, 2021).

### 2.4 fMRI image acquisition

We used the same 3 Tesla MRI Scanner (Siemens Biograph mMR) at the Lawson Health Research Institute for all participants. Functional whole-brain images of the BOLD contrast were acquired using a gradient echo T2*-weighted echo-planar-imaging sequence (TE = 30 ms, TR = 2 s, FOV = 192 mm × 192 mm, flip angle = 80°, in-plane resolution = 3 mm × 3 mm). Each volume consisted of 36 ascending interleaved slices tilted −20° from the AC-PC axis with a thickness of 3 mm and a slice gap of 1 mm. Participants’ heads were stabilized using a 32-channel head coil. These scanning parameters enabled whole-brain coverage. The experimental runs comprised 284 volumes, and T1-weighted anatomical images were obtained using a Magnetization Prepared Rapid Acquisition Gradient Echo sequence (TE = 3.03 ms, TR = 2.3 s, 192 slices, FOV = 256 mm × 256 mm).

### 2.5 fMRI preprocessing

We preprocessed all neuroimaging data following identical procedures using SPM12 within MATLAB R2020a. We discarded the first four functional volumes for each subject and then performed slice time correction to the middle slice as well as reorientation to the AC-PC axis. Using a rigid body transformation, we performed spatial alignment to the mean functional image to correct for participant movement during the scan. The functional images were also resliced. T1 anatomical images were corrected for inhomogeneity. We then used the mean functional image to co-register the functional scans to the subject-specific T1-weighted anatomical image. Co-registration was visually inspected for each subject and was manually corrected if necessary. Subsequently, we performed segmentation of all tissue types with the co-registered images using the “New Segment” method in SPM. Volumes were then spatially normalized (2 mm^3^ × 2 mm^3^ × 2 mm^3^) to the MNI standard template via application of a deformation matrix. Functional images were then smoothed via a three-dimensional isotropic 6-mm FWHM Gaussian kernel. Lastly, we performed additional motion correction using the Artifact Detection Tool (ART) software package,^2^ which computes regressors to account for outlier volumes, in addition to the six movement regressors computed during standard realignment procedures in general linear modeling. We selected the standard thresholds for outlier detection in ART (global signal threshold = 9.0 mm, absolute subject motion threshold = 2.0 mm, rotational threshold = 0.05 mm, scan-to-scan subject motion = 2.0 mm, and scan-to-scan subject rotation = 0.02 mm).

### 2.6 Statistical analyses

#### 2.6.1 First-level analysis

Separate sessions were defined for each of the neurofeedback training runs and transfer run. All task events (initial rest, instructions, fixation cross, and conditions) were modeled as blocks of brain activation and convolved with the hemodynamic response function. At this stage, functional data was high-pass filtered and serial correlations were taken into account using an autoregressive model. Additionally, we included the ART regressors as nuisance variables to account for additional movement and outlier artifacts. The three experimental conditions (*regulate*, *view*, and *neutral*) were modeled separately.

#### 2.6.2 Whole-brain neural activation analysis – second-level

We conducted a mixed-effects model, split-plot factorial 2 (Neurofeedback target region: PCC, amygdala) × 3 (Run: 1, 2, 3) × 2 (Condition: regulate, view) ANCOVA with covariates for biological sex and CAPS-5 scores within SPM12 [hereafter referred to as, “training runs ANCOVA”]. We examined the transfer run separately by conducting a mixed-effects model, split-plot factorial 2 (Neurofeedback target region: PCC, amygdala) × 2 (Condition: regulate, view) ANCOVA with the same covariates [hereafter referred to as, “transfer run ANCOVA”]. We chose to include biological sex and CAPS-5 scores as covariates in both analyses as they differed significantly between the two participant groups. We were specifically interested in investigating differential neural activation between the two participant groups while they exerted regulatory control over activity within the neurofeedback target region. Therefore, we conducted *a priori t*-tests directly comparing whole-brain activation between the two groups during the *regulate* condition. All statistical tests were corrected for multiple comparisons using a cluster-level false discovery rate (FDR) significance threshold of *p* < 0.05, *k* = 10, with an initial cluster defining threshold at *p* < 0.001, *k* = 10 (Eklund et al., 2016; Roiser et al., 2016). As the SPM software version that was utilized only permits one-sided *t*-tests, we opted for a conservative approach by adjusting the statistical thresholds (i.e., *p* < 0.05/2 = 0.025) of the one-sided *t*-tests to achieve identical results to those that would be obtained through the use of a two-sided *t*-test (Chen et al., 2018).

#### 2.6.3 Neurofeedback target downregulation analysis

To evaluate downregulation of the neurofeedback target region (i.e., neurofeedback success), we used rfxplot software to extract the event-related BOLD response (peristimulus time histogram) from the appropriate target region (i.e., PCC or amygdala during the *regulate* and *view* conditions) (Gläscher, 2009). Within the search volume, we extracted event-related BOLD responses from individual peaks for each subject and imported these values into SPSS (v.29) for statistical analyses. In rfxplot software, event-related BOLD responses are represented as the average height of the BOLD responses within a user-defined search volume and time window (Gläscher, 2009). Event-related BOLD responses are estimated using a condition-specific Finite Impulse Response (FIR) model (Gläscher, 2009). For the search volume, we used identical regional definitions as was used during participant scanning to generate the neurofeedback signal from each neurofeedback target region. For the time window, we parcellated the condition duration into temporal bins (TR = 2 s) starting at the onset of all trials belonging to a particular condition. The parameter estimate of each temporal bin within the FIR model is equivalent to that of the bin’s mean BOLD response. Thus, the outcome of the FIR model is an event-related BOLD time course for each subject.

Previously, we observed that participants were able to successfully downregulate BOLD activity within both the amygdala (Nicholson et al., 2016a, 2018) and PCC (Nicholson et al., 2021). Here, we sought to determine whether participants’ neurofeedback performance differed between the two target regions during rt-fMRI-NFB. Thus, we conducted a mixed-effects model, split-plot factorial 2 (Neurofeedback target region: PCC, amygdala) x 4 (Run: 1, 2, 3, 4) x 2 (Condition: regulate, view) repeated measures ANCOVA with covariates for biological sex and CAPS-5 scores. Subsequently, we conducted *a priori* defined independent sample *t*-tests, comparing average BOLD response between the two target regions for *regulate* and *view* conditions during each neurofeedback run.

#### 2.6.4 State changes in PTSD symptoms over neurofeedback

We assessed state changes in PTSD symptoms to traumatic stimuli after each neurofeedback run (including the transfer run), as measured by the RSDI scale. As this data was not normally distributed, we conducted non-parametric Friedman’s repeated measures ANOVAs for each RSDI subscale for the participant groups separately to measure within-group changes in state PTSD symptoms. We Bonferroni corrected our statistical threshold (*p* < 0.05/5 = 0.01) for multiple nonparametric ANOVAs. We then examined two *a priori* planned comparisons between RSDI subscale scores at different time points – post-run 1 vs. post-run 3 and post-run 1 vs. post-run 4 – using non-parametric tests for related samples (Wilcoxon signed-ranks test). Finally, we compared RSDI subscale scores between participant groups after each neurofeedback run (including the transfer run) using Mann–Whitney U tests. As an additional precaution, we performed a similar between-group comparison using a Quade’s rank-transformed ANCOVA with CAPS-5 scores as a covariate which yielded identical results. Please note, within-group changes on RSDI subscale scores for the PCC group have been reported elsewhere (Nicholson et al., 2021; Lieberman et al., 2023).

## 3 Results

### 3.1 Downregulation of neurofeedback target brain region

**Figure 2:**
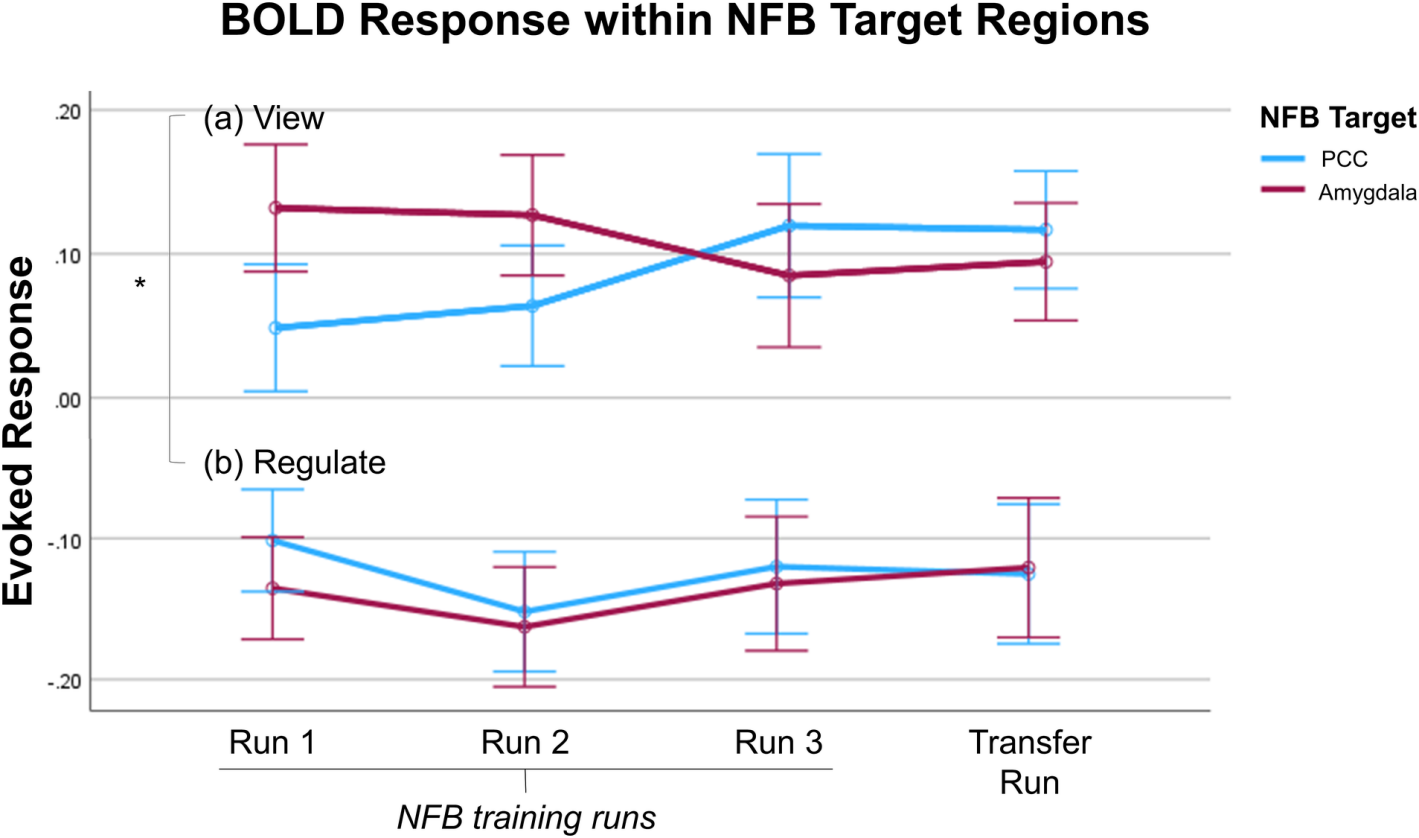
Average event-related BOLD response in the NFB target regions – PCC (blue) or amygdala (red) – is shown for the **(A)** *view* and **(B)** *regulate* conditions during the three NFB training runs and transfer run. For both the PCC and amygdala, average event-related BOLD response is significantly lower during *regulate* as compared to *view* conditions for each NFB training run and the transfer run. There were no significant differences in the average event-related BOLD response between the two NFB target regions during *regulate* or *view* in any individual NFB run. The x-axis of the graph indicates the NFB run; the y-axis indicates the average event-related BOLD response (peristimulus time histogram) across the entire duration of the condition. Error bars indicate standard error of the mean. PCC, posterior cingulate cortex; NFB, neurofeedback.

Previously, we found that individuals with PTSD were able to significantly downregulate BOLD activity within the PCC (Nicholson et al., 2021) and amygdala (Nicholson et al., 2016a) during *regulate* as compared to *view* conditions for all three neurofeedback training runs, as well as the transfer run. Additionally, for both participant groups, activity within the target region during *regulate* did not significantly differ when directly comparing across neurofeedback runs (Nicholson et al., 2016a, 2021). Here, within the ANCOVA examining the down regulation of the neurofeedback target regions, we observed a significant main effect of condition [*F*(1, 24) = 10.33, η^2^ = 0.301, *p* = 0.004]. We also observed non-significant main effects of run [*F*(3, 72) = 0.668, η^2^ = 0.027, *p* = 0.575] and group [*F*(1, 24) = 0.030, η^2^ = 0.001, *p* = 0.864], with non-significant interaction effects. Follow-up independent sample *t*-tests revealed that there was no significant difference in the average event-related BOLD response within the target region between the two groups during *regulate* or *view* in any individual neurofeedback run [regulate, run 1: *t*(26) = 0.197, *p* = 0.846, Cohen’s *d* = 0.074; regulate, run 2: *t*(26) = 0.342, *p* = 0.735, Cohen’s *d* = 0.129; regulate, run 3: *t*(26) = 0.637, *p* = 0.530, Cohen’s *d* = 0.241; regulate, run 4: *t*(26) = 0.794, *p* = 0.435, Cohen’s d = 0.300; view, run 1: *t*(26) = −1.11, *p* = 0.278, Cohen’s d = −0.419; view, run 2: *t*(26) = −0.064, *p* = 0.949, Cohen’s d = −0.024; view, run 3: *t*(26) = 0.045, *p* = 0.965, Cohen’s d = 0.017; view, run 4: *t*(26) = −0.645, *p* = 0.525, Cohen’s *d* = −0.244; Figure 2]. Importantly, this shows that both participant groups exhibited similar activation within the target region in response to the trauma-related words (i.e., the *view* comparisons) and were similarly able to downregulate activation within their target region (i.e., the *regulate* comparisons). Therefore, neural activation results can be confidently attributed to the differential effect of the selected neurofeedback target region for each participant group rather than resulting from possible group differences in trauma-related responses or success in regulating the neurofeedback target (Sorger et al., 2019).

### 3.2 Whole-brain neural activation analysis

Our neurofeedback training runs ANCOVA revealed significant main effects for neurofeedback target and condition (Table 2). The main effect of neurofeedback target showed significant clusters within the bilateral cuneus/precuneus/primary visual cortex, the left superior parietal lobule, and the left occipital pole. The main effect of condition showed significant clusters within the left superior/middle frontal gyrus, the left angular gyrus, and the left precuneus. There was a non-significant main effect of run and non-significant interaction effects (Table 2).

**Table 2:** 2 (Group) × 3 (NFB Run) × 2 (Condition) – training runs ANCOVA.

				MNI coordinate					
Comparison	Brain region	H	*k*	*x*	*y*	*z*	*F*-Stat.	*Z-*Score	*p*-FDR
Main effect of NFB target	Cuneus/precuneus/primary visual cortex	Right	1,127	14	−72	20	34.38	5.44	<0.001
	Cuneus/precuneus/primary visual cortex	Left	97	−18	−68	10	15.89	3.71	0.03
	Superior parietal lobule	Left	175	−22	−60	38	24.26	4.6	0.004
	Occipital pole	Left	118	−14	−100	18	18.39	4	0.018
Main effect of run	*ns*								
Main effect of condition	Superior/middle frontal gyrus	Left	132	−18	26	44	21.87	4.37	0.022
	Angular gyrus	Left	144	−50	−72	30	18.7	4.03	0.022
	Precuneus	Left	110	−10	−54	38	16.52	3.79	0.033
NFB target × run	*ns*								
NFB target × condition	*ns*								
Run × condition	*ns*								
NFB target × run × condition	*ns*								

Results of the split-plot factorial 2 (group) by 3 (NFB run) by 2 (condition) ANCOVA evaluated at the FDR-cluster corrected threshold for multiple comparisons (*p* < 0.05, *k* = 10). The comparison, brain region, hemisphere of the region (H), cluster size (*k*), MNI coordinates (*x*,*y*,*z*), *F*-statistic (*F*-Stat.), *Z*-Score, and significance (*p*-FDR) of each significant cluster are included as columns. *ns*, not significant; NFB, neurofeedback.

**Figure 3:**
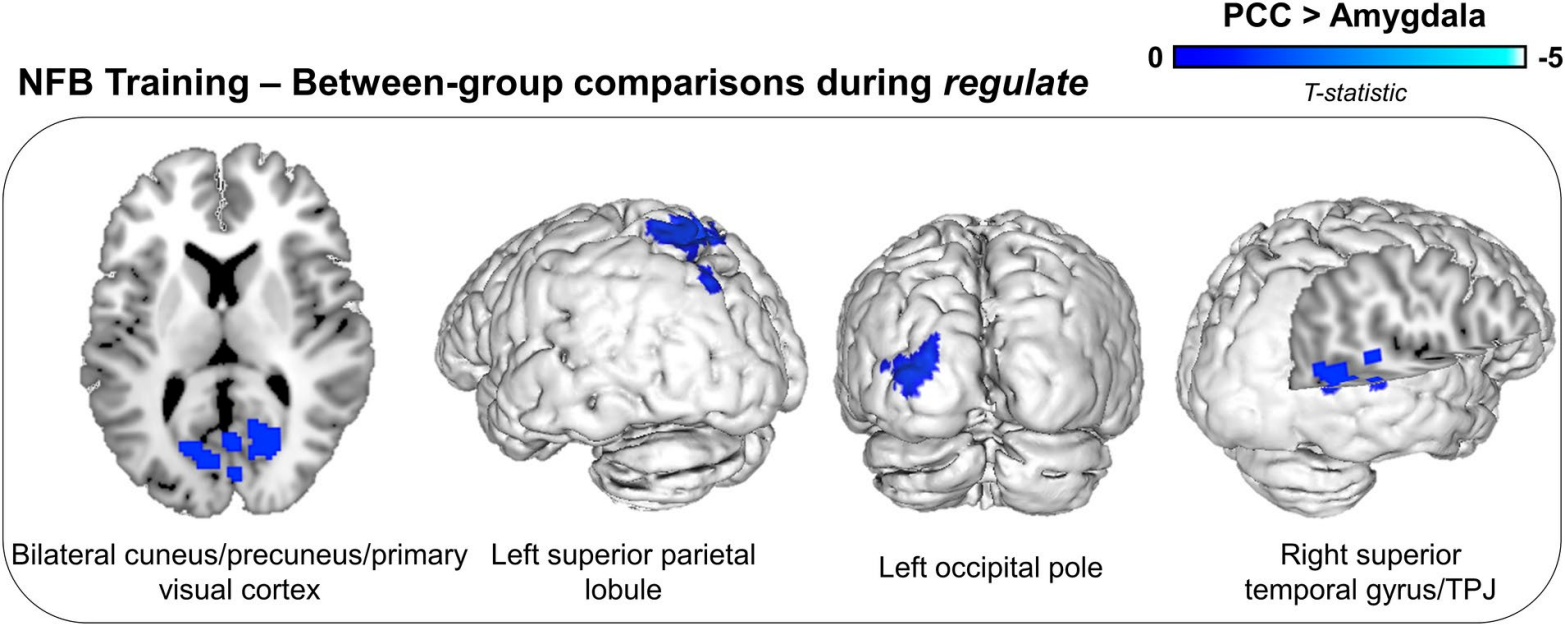
Between-group differences in neural activity during NFB downregulation of the target region (PCC or amygdala). Displayed brain regions show concomitant decreases in neural activity for the PCC as compared to the amygdala group during downregulation across NFB training runs. Results are corrected for multiple comparisons using a cluster-level false discovery rate (FDR) significance threshold of *p* < 0.025, *k* = 10. TPJ, temporoparietal junction.

For the amygdala as compared to the PCC group, we did not observe any significant unique (i.e., over and above that of the PCC group) decreases in neural activity during the *regulate* condition (Table 3). Conversely, for the PCC as compared to the amygdala group, we observed widespread whole-brain decreases in activity within the bilateral cuneus/precuneus/primary visual cortex, the left superior parietal lobule, the left occipital pole, and the right superior temporal gyrus/temporoparietal junction (TPJ) (Table 3; Figure 3).

**Table 3:** Neurofeedback training – between-group comparisons during *regulate.*

					MNI coordinate					
Comparison	Condition	Brain region	H	*k*	*x*	*y*	*z*	*T*-Stat.	*Z-*Score	*p*-FDR
Amygdala < PCC	Regulate	*ns*								
PCC < Amygdala	Regulate	Cuneus/precuneus/primary visual cortex	Bilateral	1,643	14	−74	22	5.87	5.57	<0.001
		Superior parietal lobule	Left	856	−22	−60	38	5.12	4.91	<0.001
		Occipital pole	Left	198	−20	−90	8	4.24	4.12	0.007
		Superior temporal gyrus/TPJ	Right	194	56	−38	16	3.99	3.89	0.007

Results of the *a-priori* between-group comparisons for the *regulate* condition during the NFB training runs evaluated at the FDR-cluster corrected threshold for multiple comparisons (*p* < 0.025, *k* = 10). The comparison, condition, brain region, hemisphere of the brain region (H), cluster size (*k*), MNI coordinates (*x*,*y*,*z*), *T*-statistic (*T*-Stat.), *Z*-Score, and significance (*p*-FDR) of each significant cluster are included as columns. *ns*, not significant; PCC, posterior cingulate cortex; TPJ, temporoparietal junction.

**Figure 4:**
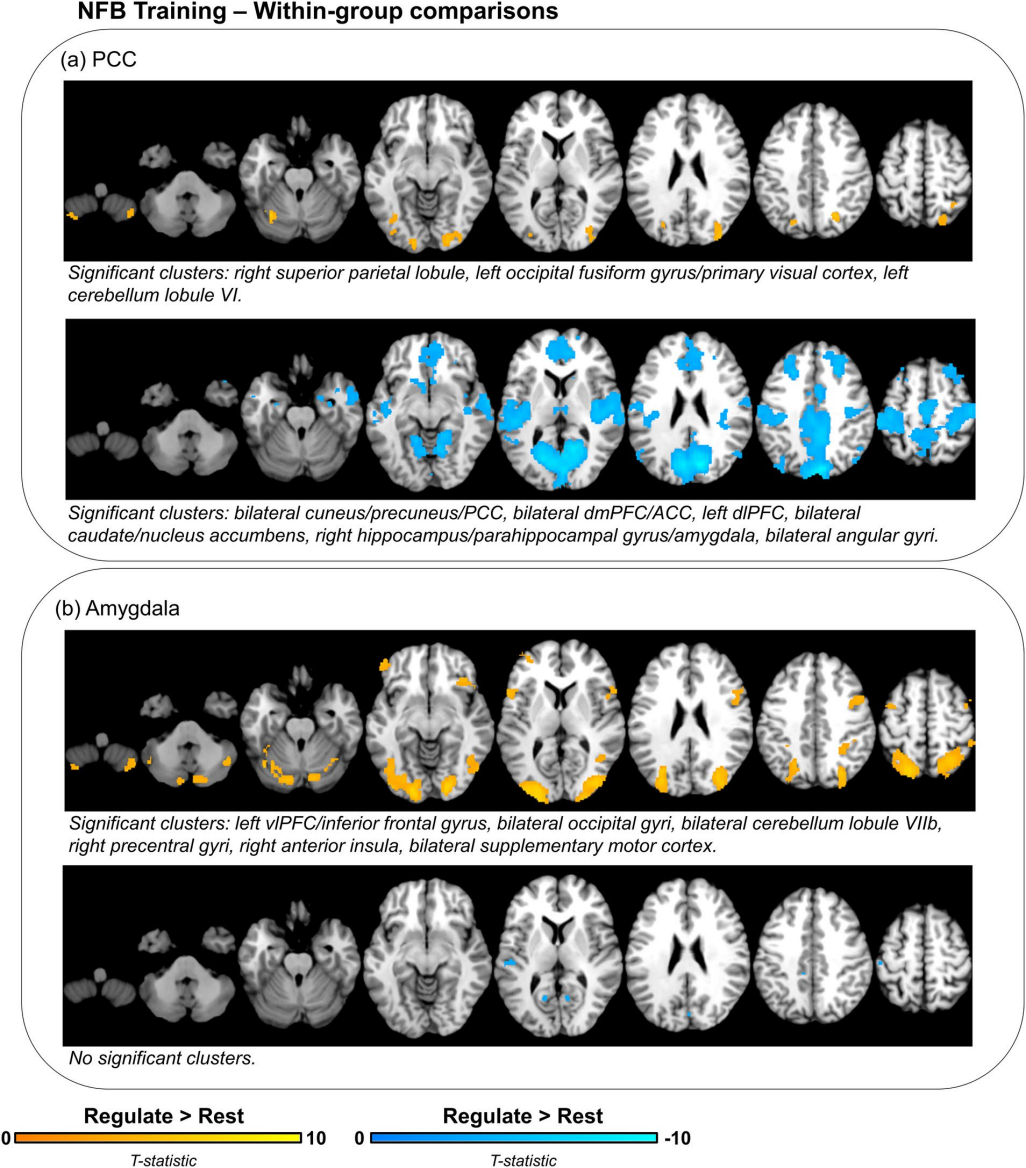
Within-group differences in neural activity during NFB downregulation of target region (PCC or amygdala) as compared to rest. Displayed brain regions show concomitant increases (yellow) or decreases (blue) in neural activity for the **(A)** PCC and **(B)** amygdala groups during *regulate* as compared to rest. Brain images are thresholded with an initial cluster defining threshold at *p* < 0.001, *k* = 10. Significant clusters are identified using a cluster-level false discovery rate (FDR) significance threshold of *p* < 0.025, *k* = 10. PCC, posterior cingulate cortex; vlPFC, ventrolateral prefrontal cortex; dmPFC, dorsomedial prefrontal cortex; ACC, anterior cingulate cortex; dlPFC, dorsolateral prefrontal cortex.

Importantly, in conducting between-group comparisons of neural activity, any changes in neural activity that are shared by both groups would not be observable. As such, we conducted follow-up comparisons of neural activity during the *regulate* condition as compared to rest for each group separately (Table 4; Figure 4). During *regulate* as compared to rest, the PCC group showed widespread whole-brain decreases in activity including a large bilateral posterior cluster extending across the cuneus, precuneus, primary visual cortex, and PCC, as well as significant clusters within the dmPFC/ACC, left dlPFC, bilateral caudate/nucleus accumbens, right hippocampus/parahippocampal gyrus/amygdala, and bilateral angular gyri (Figure 4A – bottom panel). The PCC group only showed increased activity in posterior regions of the brain with significant clusters within the right superior parietal lobule, the left occipital gyrus/primary visual cortex, and the left cerebellum lobule VI (Figure 4A – top panel). Conversely, during *regulate* as compared to rest, the amygdala group did not show any significant whole-brain decreases in activity (Figure 4B – bottom panel). However, this group showed increased activity in several brain regions including the left vlPFC/inferior frontal gyrus, bilateral occipital gyri, bilateral cerebellum lobule VIIb, right precentral gyri, right anterior insula, and the bilateral supplementary motor cortex (Figure 4B – top panel).

**Table 4:** Neurofeedback training – within-group comparisons.

					MNI coordinate					
NFB target	Condition	Brain region	H	*k*	*x*	*y*	*z*	*T*-Stat.	*Z-*Score	*p*-FDR
PCC	Regulate > Rest	Superior parietal lobule	Right	994	32	−78	14	7.18	6.66	<0.001
		Occipital fusiform gyrus/primary visual cortex	Left	239	−14	−90	−10	5.84	5.54	0.001
		Cerebellum lobule VI	Left	142	−30	−62	−22	5.82	5.53	0.013
Amygdala	Regulate > Rest	Occipital gyrus	Right	4,554	18	−88	−2	7.87	7.21	<0.001
		Occipital gyrus	Left	4,942	−16	−92	−8	7.6	6.99	<0.001
		Cerebellum lobule VIIb	Left	102	−22	−72	−48	5.76	5.47	0.018
		Anterior insula	Right	449	36	28	−8	5.31	5.08	<0.001
		Inferior frontal gyrus	Left	261	−48	46	0	5.14	4.93	<0.001
		Precentral gyrus	Right	295	36	−4	44	4.77	4.6	<0.001
		Cerebellum lobule VIIb	Right	157	32	−66	−48	4.76	4.59	0.005
		vlPFC/Inferior frontal gyrus	Left	117	−54	14	8	4.75	4.58	0.014
		Supplementary motor cortex	Bilateral	108	4	2	64	4.71	4.55	0.016
PCC	Regulate < Rest	Cuneus/precuneus/primary visual cortex/PCC	Bilateral	24,962	12	−64	14	10.89	>8	<0.001
		dmPFC/ACC	Bilateral	3,725	24	28	40	6.6	6.18	<0.001
		dlPFC	Left	693	−22	26	42	6.32	5.95	<0.001
		Caudate/nucleus accumbens	Bilateral	147	−8	6	−4	4.71	4.55	0.01
		Hippocampus/parahippocampal gyrus/amygdala	Right	195	28	−18	−16	4.54	4.39	0.003
		Angular gyrus	Left	126	−38	−78	38	4.47	4.33	0.016
		Angular gyrus	Right	291	42	−70	36	4.12	4	<0.001
Amygdala	Regulate < Rest	*ns*								

Results of the follow-up within-group comparisons between *regulate* and rest during the NFB training runs evaluated at the FDR-cluster corrected threshold for multiple comparisons (*p* < 0.025, *k* = 10). The NFB target, condition, brain region, hemisphere of the brain region (H), cluster size (*k*), MNI coordinates (*x*,*y*,*z*), *T*-statistic (*T*-Stat.), *Z*-Score, and significance (*p*-FDR) of each significant cluster are included as columns. *ns*, not significant; PCC, posterior cingulate cortex; vlPFC, ventrolateral prefrontal cortex; dmPFC, dorsomedial prefrontal cortex; ACC, anterior cingulate cortex; dlPFC, dorsolateral prefrontal cortex.

Our transfer run ANCOVA did not reveal any significant main effects or interactions between factors. For the transfer run, *a priori* planned comparisons between the two participant groups during the *regulate* condition yielded non-significant results.

### 3.3 State changes in PTSD symptoms over neurofeedback

In summary, when assessing state changes in PTSD symptoms, we observed clear differences in terms of within-group results for the PCC and amygdala groups. As previously published, PCC downregulation was found to show a significant main effect of run for the nonparametric ANOVA investigating reliving [*χ*^2^(3) = 11.49, *p* = 0.009] and distress [*χ*^2^(3) = 13.79, *p* = 0.003] symptoms, and non-significant effects for the other RSDI subscales [physical reactions: *χ*^2^(3) = 4.70, *p* = 0.195; emotional numbing: *χ*^2^(3) = 2.26, *p* = 0.520; dissociation: *χ*^2^(3) = 2.29, *p* = 0.515] when controlling for multiple comparisons (Nicholson et al., 2021). Wilcoxon signed-rank tests revealed lower reliving scores during run 3 versus run 1 (*p* = 0.016) and lower distress scores during runs 3 (*p* = 0.010) and 4 (*p* = 0.013) versus run 1 for the PCC group (Nicholson et al., 2021; Table 5). By contrast, amygdala downregulation was not found to show a significant main effect of run for any of the nonparametric ANOVAs that were conducted for each of the RSDI subscales [reliving: *χ*^2^(3) = 9.21, *p* = 0.027; distress: *χ*^2^(3) = 4.98, *p* = 0.173; physical reactions: *χ*^2^(3) = 10.24, *p* = 0.017; emotional numbing: *χ*^2^(3) = 0.240, *p* = 0.971; dissociation: *χ*^2^(3) = 0.241, *p* = 0.971] when controlling for multiple comparisons. Wilcoxon signed-rank tests did not reveal any significant differences in RSDI scores between runs (i.e., run 1 vs. run 3; run 1 vs. run 4) for the amygdala group. When directly comparing the two groups during each run using Mann–Whitney U tests, there were no significant differences observed for any of the RSDI subscale scores (Table 6).

**Table 5:** Reliving and distress symptoms during NFB.

	NFB training: Run 1		NFB training: Run 2		NFB training: Run 3		NFB transfer: Run 4		Run 1 vs Run 3			Run 1 vs Run 4		
NFB Target	Mean	SD	Mean	SD	Mean	SD	Mean	SD	*Z-*Score	*p*	Effect Size (*r*)	*Z-*Score	*p*	Effect Size (*r*)
*Reliving*														
PCC	2.71	1.86	2.29	1.86	1.86	1.83	1.86	1.79	−2.401	0.016*	−0.454*	−1.981	0.050	−0.374
Amygdala	2.36	1.55	1.86	1.96	1.57	1.65	1.57	1.95	−1.930	0.054	−0.364	−1.826	0.068	−0.345
*Distress*														
PCC	2.93	1.21	2.79	1.72	2.07	1.44	2.07	1.54	−2.588	0.010*	−0.489*	−2.489	0.013*	−0.470*
Amygdala	2.50	2.03	2.57	1.95	2.36	1.55	1.86	1.83	−0.439	0.660	−0.083	−1.224	0.221	−0.231

The left side of the table shows the mean and SD results for reliving and distress symptoms (as measured by RSDI) for the PCC and amygdala groups after each of the neurofeedback training runs and transfer run (i.e., Run 4). The right side of the table shows results of the a priori comparisons between reliving and distress symptoms for Run 1 as compared to Runs 3 and 4 using non-parametric tests for related samples (Wilcoxon signed-ranks test). The *Z*-Score, significance (*p*) and effect size (r) of each comparison are included as columns. Asterisks indicate significant results. PCC, posterior cingulate cortex; NFB, neurofeedback; SD, standard deviation.

**Table 6:** Between-group comparisons of PTSD symptoms during NFB.

		PCC vs Amygdala			
Run	Group	Mean rank	*Z-*Score	*p*	Effect size (*r*)
*Reliving*					
Run 1	PCC	15.39	−0.588	0.556	−0.111
	Amygdala	13.61			
Run 2	PCC	15.54	−0.683	0.495	−0.129
	Amygdala	13.46			
Run 3	PCC	14.96	−0.310	0.757	−0.059
	Amygdala	14.04			
Run 4 (transfer)	PCC	15.39	−0.602	0.547	−0.114
	Amygdala	13.61			
*Distress*					
Run 1	PCC	15.61	−0.723	0.470	−0.137
	Amygdala	13.39			
Run 2	PCC	14.75	−0.165	0.869	−0.031
	Amygdala	14.25			
Run 3	PCC	13.75	−0.498	0.619	−0.094
	Amygdala	15.25			
Run 4 (transfer)	PCC	15.29	−0.515	0.606	−0.097
	Amygdala	13.71			
*Physical reactions*					
Run 1	PCC	13.50	−0.654	0.513	−0.124
	Amygdala	15.50			
Run 2	PCC	14.61	−0.070	0.944	−0.013
	Amygdala	14.39			
Run 3	PCC	14.71	−0.140	0.888	−0.026
	Amygdala	14.29			
Run 4 (transfer)	PCC	14.86	−0.235	0.814	−0.044
	Amygdala	14.14			
*Emotional numbing*					
Run 1	PCC	15.36	−0.568	0.570	−0.107
	Amygdala	13.64			
Run 2	PCC	14.89	−0.259	0.796	−0.049
	Amygdala	14.11			
Run 3	PCC	14.46	−0.023	0.981	−0.004
	Amygdala	14.54			
Run 4 (transfer)	PCC	15.14	−0.423	0.672	−0.080
	Amygdala	13.86			
*Dissociation*					
Run 1	PCC	14.29	−0.142	0.887	−0.027
	Amygdala	14.71			
Run 2	PCC	14.25	−0.170	0.865	−0.032
	Amygdala	14.75			
Run 3	PCC	13.96	−0.360	0.719	−0.068
	Amygdala	15.04			
Run 4 (transfer)	PCC	15.00	−0.335	0.737	−0.063
	Amygdala	14.00			

Results of the between-group comparisons of PTSD symptoms (as measured by RSDI) after each run using Mann–Whitney U Tests. The run, group, mean rank, *Z*-Score, significance (*p*), and effect size (*r*) are included as columns. NFB, neurofeedback.

## 4 Discussion

### 4.1 Overview

The complexity of neurobiological alterations associated with PTSD poses a challenge for selecting an optimal target region for the regulation of neural activity using rt-fMRI-NFB. In the present analysis, we conducted a direct comparison of whole-brain activation patterns and changes in symptoms between two groups of PTSD participants who were trained to downregulate activity in clinically relevant target regions – either the amygdala or the PCC – using rt-fMRI-NFB. We observed significant differences in whole-brain activity during the *regulate* condition depending on the neurofeedback target region. For the PCC group as compared to the amygdala group, there were whole-brain decreases in activity within the bilateral cuneus/precuneus/primary visual cortex, the left superior parietal lobule, the left occipital pole, and the right superior temporal gyrus/TPJ (Figure 3). In contrast, for the amygdala group as compared to the PCC group, there were no unique (i.e., over and above that of the PCC group) decreases in neural activity. Interestingly, we observed clear differences in clinical outcomes between participants in the two groups with acute decreases in state PTSD symptoms observed for the PCC group only. Indeed, downregulation of amygdala activity via rt-fMRI-NFB did not lead to improvements in state PTSD symptoms among participants, whereas downregulation of PCC activity was associated with reduced reliving and distress symptoms (Nicholson et al., 2018, 2021; Lieberman et al., 2023). As previously reported, participants were able to successfully downregulate activity within both of these target brain regions (Nicholson et al., 2016a, 2021). Importantly, in the present analysis, we found that there was no significant difference between the two participant groups in terms of their neural response to trauma-related words within the target region or their ability to downregulate activity within their respective target brain regions, thus serving as a critical control for the between-group neural activation results. Moreover, participants in both groups reported using similar regulatory strategies including mindfulness-based techniques, positive self-talk, and the use of visual imagery.

### 4.2 Differential neural activation

Significant differences in neural activity based on the target region for rt-fMRI-NFB (during the *regulate* condition) were observed in the present analysis. For the PCC group as compared to the amygdala group, downregulation of the target region was concomitantly associated with whole-brain decreases in neural activity, with a particular focus toward posterior areas. Specifically, participants in the PCC as compared to the amygdala group, showed simultaneous downregulation within the bilateral cuneus/precuneus/primary visual cortex, the left superior parietal lobule, the left occipital pole, and the right superior temporal gyrus/TPJ (Figure 3), all of which have been linked to PTSD psychopathology (Lanius et al., 2002; Hopper et al., 2007b; Yin et al., 2011; Nilsen et al., 2016; Clancy et al., 2017; Kunimatsu et al., 2020; Sheynin et al., 2020). The precuneus is closely linked - both anatomically and functionally - with the PCC and comprises a critical node within the posterior DMN (Cavanna and Trimble, 2006; Fransson and Marrelec, 2008; Raichle, 2015), in which PTSD-associated alterations have been shown to be related to traumatic/negative autobiographical memories, dysregulated self-referential processing and altered social cognition (Bluhm et al., 2009; Daniels et al., 2010; Lanius, 2015; Tursich et al., 2015; Akiki et al., 2017; Fenster et al., 2018; Hinojosa et al., 2019; Lanius et al., 2020). Indeed, hyperactivity within the precuneus specifically has been associated with the presentation of trauma-related stimuli among individuals with PTSD (Ramage et al., 2013; Sartory et al., 2013; Thome et al., 2020) and negatively correlated with symptom improvements following treatment (Ke et al., 2016). The cuneus, primary visual cortex and occipital pole all lie within the occipital lobe and are necessary for processing visual information and contribute to the formation and perception of visual imagery (Le Bihan et al., 1993; Klein et al., 2000; Thiebaut de Schotten et al., 2014; Palejwala et al., 2021). In the context of PTSD, visual cortex hyperactivity has been shown to be positively correlated with symptom severity (Zhu et al., 2014, 2015; Neumeister et al., 2017; Suo et al., 2020). Indeed, hyperactivity (Rauch, 1996; Hendler et al., 2003; Todd et al., 2015; Neumeister et al., 2017) and elevated connectivity (Neumeister et al., 2017; Rabellino et al., 2018b; Suo et al., 2020) within the visual cortex during trauma-related cue exposure may specifically correspond to the visual component of PTSD reliving and reexperiencing symptoms. Interestingly, decreased neural activity was also observed within brain regions that are closely linked to PTSD-associated alterations in other sensory domains and cognitive processing.

In the present neural activation analysis, decreased neural activity was also observed in the posterior region of the right superior temporal gyrus/TPJ, for the PCC as compared to the amygdala group. The temporal gyrus displays increased activation in response to trauma-related stimuli and is positively correlated to PTSD symptoms of avoidance and dissociation (Lanius et al., 2002; Hopper et al., 2007b; Nilsen et al., 2016). The TPJ is involved in multisensory (i.e., visual, vestibular, and somatosensory) integration, bodily self-consciousness, and embodiment (Arzy et al., 2006; Blanke, 2012; Igelström et al., 2015), processes whose impairment may underlie PTSD symptoms related to dissociation, emotion constriction/detachment, and altered interoception/exteroception (Hopper et al., 2007b; Lanius et al., 2012; Lanius, 2015; Harricharan et al., 2016, 2021; Rabellino et al., 2020; Kearney and Lanius, 2022). The superior temporal gyrus/TPJ are also involved in reperesenting one’s peripersonal space (i.e., the area surrounding the body where one can reach or be reached by external entities, including objects or other individuals) (Bernasconi et al., 2018; Rabellino et al., 2020). It has been suggested that in PTSD one’s peripersonal space is likely to be larger in size (for defensive purposes) and more rigid (less able to modify the body schema in response to shifting multisensory inputs) (Rabellino et al., 2020). Interestingly, neuroimaging data suggest the existence of a specific neural network involving the PCC, TPJ, and intraparietal sulcus, which together generate one’s sense of self-location, a fundamental aspect of bodily self-consciousness (Park and Blanke, 2019). Hence, simultaneous downregulation of TPJ and PCC activity during trauma provocation may suggest a recalibration of the neural system that enables proper bodily self-consciousness functionality and multisensory integration, and thereby may reduce PTSD symptoms.

The left superior parietal lobule, which also showed decreased activity in the present neural activation analysis in the PCC as compared to the amygdala group, is pivotal in several sensory and cognitive processes, including somatosensory and visuomotor integration (Culham and Valyear, 2006; Iacoboni, 2006), motor learning (Weiss et al., 2003; Wenderoth et al., 2004) and exerting top-down attentional control (Behrmann et al., 2004). As a multimodal sensory integration region, the superior parietal lobule also helps to construct an awareness of one’s internal state, body schema, and relation to external space (Pearson, 2009). In this regard, the superior parietal lobule shares functional overlap with the temporal gyrus/TPJ in contributing to body part localization (Felician et al., 2004), bodily self-consciousness (Tsakiris et al., 2007), and representing one’s peripersonal space (Lloyd et al., 2006; Gallivan et al., 2011), processes which are often altered in PTSD (Rabellino et al., 2016, 2020; Rabellino et al., 2018a). During a memory retrieval paradigm, participants with PTSD showed hyperconnectivity between the posterior DMN and several sensorimotor network hubs (Kearney et al., 2023), including the left superior parietal lobule. This may reflect the integration of trauma-related sensorimotor imprints with autobiographical memory, thereby contributing to the vividness of reexperiencing and/or reliving of symptoms (van der Kolk and Fisler, 1995; Brewin et al., 1996; Kearney and Lanius, 2022). The superior parietal lobule is also a critical node within the dorsal attention network and is thought to play a key role in orienting visuospatial attention (Corbetta et al., 1993, 1995; Corbetta and Shulman, 2002; Lanssens et al., 2020). Altered connectivity of the dorsal attention network as a whole has been found among individuals with PTSD (Russman Block et al., 2017; Evans et al., 2022). Disrupted attentional processes, such as response inhibition and attention regulation, may represent a critical mechanism underlying PTSD symptom development and relate to PTSD symptom severity due to difficulties in disengaging from threat/defence processing and reorienting attention to task-relevant stimuli (Pineles et al., 2007, 2009; Aupperle et al., 2012; Russman Block et al., 2017; Evans et al., 2022). Taken together, decreased activity within the posterior DMN (i.e., precuneus), visual cortex (i.e., cuneus/primary visual cortex and occipital lobe), temporal gyrus/TPJ, and superior parietal lobule, may indicate that individuals with PTSD regulating activity within the PCC, as compared to the amygdala, show greater control over trauma-related autobiographical memory, emotion, and multisensory processing. Indeed, these findings may help make sense of the fact that only the PCC group showed improvements in state PTSD symptoms (i.e., reliving and distress) with rt-fMRI-NFB training during the trauma provocation paradigm (Nicholson et al., 2016a, 2018).

Conversely, for the amygdala group as compared to the PCC group, neurofeedback training was not associated with any significant unique (i.e., over and above that of the PCC group) decreases in neural activity during the *regulate* condition. This suggests that downregulating the amygdala, as compared to the PCC, does not result in unique regulatory decreases in neural activity elsewhere in the brain. Indeed, this finding raises a critical point for consideration. In conducting comparisons of neural activity on the basis of the neurofeedback target region, any changes in neural activity that were common to both groups would not be observable. As such, we conducted supplemental comparisons of neural activity during the regulate condition as compared to rest for each group separately. For *regulate* as compared to rest, there were significant whole-brain increases in neural activity – including significant clusters in the left vlPFC/inferior frontal gyrus, bilateral occipital gyri, bilateral cerebellum lobule VIIb, right precentral gyrus, right anterior insula, and bilateral supplementary motor cortex – for the amygdala group (Figure 4B – top panel). However, there were no significant decreases in neural activity (i.e., simultaneous downregulation) for the amygdala group during *regulate* as compared to rest (Figure 4B – bottom panel). Conversely, for the PCC group, during *regulate* as compared to rest, there were widespread whole-brain decreases in neural activity – including significant clusters in the bilateral cuneus/precuneus/primary visual cortex/PCC, left dlPFC, bilateral caudate/nucleus accumbens, right hippocampus/parahippocampal gyrus/amygdala, bilateral dmPFC/ACC, and bilateral angular gyri (Figure 4A – bottom panel). Significant increases in neural activity for the PCC group during *regulate* as compared to rest were restricted to posterior brain regions, including the right superior parietal lobule, left occipital gyrus/primary visual cortex, and the left cerebellum lobule VI (Figure 4A – top panel). Interestingly, these results indicate unique neural mechanisms – largely either increased or decreased concomitant whole-brain neural activity – that are associated with downregulation of the amygdala or PCC, respectively. Taken together, downregulating the PCC using rt-fMRI-NFB appears to be more strongly associated with whole-brain decreases in neural activity, whereas downregulating the amygdala is associated with persistent activation in several brain regions.

Accumulating evidence from several previous independent analyses by our group, in which we examined each neurofeedback target region separately, supports the notion that distinct neural mechanisms are associated with rt-fMRI-NFB downregulation of the amygdala and PCC (Nicholson et al., 2016a, 2018, 2021; Lieberman et al., 2023). More specifically, we previously found that during *regulate* as compared to *view* conditions, amygdala downregulation was associated with concomitant increased neural activity within the PFC (i.e., dlPFC, vlPFC) and increased bidirectional amygdala-PFC (i.e., dlPFC, dmPFC) connectivity (Nicholson et al., 2016a). Moreover, amygdala downregulation was associated with dynamic changes in intrinsic connectivity networks, where there was increased recruitment of the left CEN (including the bilateral dlPFC) (Nicholson et al., 2018). As such, the involvement of prefrontal cortex emotion regulation regions appears to be of primary importance for amygdala downregulation using rt-fMRI-NFB. On the other hand, we previously showed in a separate analysis that during *regulate* as compared to *view* conditions, PCC downregulation was associated with simultaneous decreases in neural activity within several brain regions involved in PTSD psychopathology – i.e., the dmPFC, postcentral gyrus, amygdala/hippocampus, cingulate cortex, and temporal pole/gyri (Nicholson et al., 2021) – as well as increased PCC connectivity with the dmPFC, vmPFC, posterior insula, and amygdala (Lieberman et al., 2023). Thus, in the context of PTSD, the neural mechanisms associated with PCC downregulation appear to primarily involve the reintegration of functionally segregated posterior and anterior DMN structures and may also result in the concomitant regulation of brain regions involved in emotion generation/processing (i.e., amygdala, mid-cingulate) and embodiment (i.e., insula). Taken together, despite both being of significant clinical relevance, downregulating the PCC and amygdala may recruit distinct neural mechanisms associated with emotion regulation. Indeed, while the involvement of prefrontal cortex brain regions in emotion regulation is well established (Rauch et al., 2006; Lanius et al., 2010; Shin and Liberzon, 2010; Aupperle et al., 2012; Pitman et al., 2012; Admon et al., 2013; Ronzoni et al., 2016), emerging neurobiological evidence is beginning to identify the critical role of the DMN and PCC in facilitating emotion regulation as well (Lanius et al., 2010; Zhou et al., 2012; Garrison et al., 2013a,b; Fresco et al., 2017; Garrett et al., 2019; Sheynin et al., 2020; Messina et al., 2021). For example, PCC regulation has been associated with emotional acceptance (Messina et al., 2021), mindfulness-based meditation (Garrison et al., 2013a,b), and reduced PTSD symptoms following trauma-focused cognitive behavioral therapy (Garrett et al., 2019). Together with the current findings, this suggests that the PCC/DMN may warrant inclusion within neural mechanistic models of emotion regulation in PTSD. In addition to its role in emotion regulation, further consideration of the PCC through the lens of network-level neuroscience, may help in understanding why it may serve as a particularly effective neurofeedback target region.

### 4.3 Network-level neuroscience

Within the vast network of neuronal connections that comprise the human brain (i.e., the connectome), certain network elements possess a relatively greater number of connections thus marking them as putative network hubs (Bassett and Bullmore, 2006; van den Heuvel and Sporns, 2013; Bassett and Sporns, 2017; Oldham and Fornito, 2019). Network hubs are critical in that they facilitate the integration between functionally specialized and anatomically distributed brain regions to enable higher-level cognitive and mental processing (Bassett and Bullmore, 2006; van den Heuvel and Sporns, 2013; Bassett and Sporns, 2017; Oldham and Fornito, 2019). While both the amygdala and PCC constitute critical hubs within each of their networks – the SN and DMN, respectively (Zuo et al., 2010; Menon, 2011, 2020) – research indicates that the PCC may be a particularly effective neurofeedback target region, from a network neuroscience-level perspective. Studies using both anatomical (Hagmann et al., 2008) and functional connectivity (Buckner et al., 2009; Tomasi and Volkow, 2011) measures have identified regions in the DMN, including the PCC specifically, as having the highest global brain connectivity values. Additionally, analysis of structural brain networks revealed that hubs of regional and global controllability – or the ability to influence subsequent neurophysiological dynamics in other regions – are preferentially located in the DMN (Gu et al., 2015). The PCC in particular plays a heterogeneous role in cortical dynamics by communicating with multiple large-scale brain networks (Leech et al., 2012; Leech and Smallwood, 2019). Such highly distributed patterns of cortical dynamics may underlie the diverse functionality of the PCC, which can be broadly categorized into task-negative (e.g., deactivation during complex external tasks), representational (e.g., self-related and social cognition), and dynamic (e.g., performance monitoring and exploration, regulation of neural dynamics) roles (Leech and Smallwood, 2019). Owing to its highly distributed functional connectivity, and the fact that it is a hub for regional and global controllability, the PCC is well positioned to regulate homeostatic balance between large-scale brain networks (Leech and Smallwood, 2019). In alignment with this proposed functionality of the PCC, EEG-NFB targeting regulation of alpha oscillations – which are correlated with PCC activation (Mantini et al., 2007; Jann et al., 2009; Clancy et al., 2020) – may promote the homeostatic normalization (i.e., self-tuning neuroplasticity) of pathological large-scale brain dynamics (Ros et al., 2014, 2017; Nicholson et al., 2023). Indeed, among individuals with PTSD, alpha-rhythm EEG-NFB was shown to recalibrate altered spontaneous long-range temporal correlations, where the degree of inter-individual recalibration was positively correlated with reduced hyperarousal symptoms (Ros et al., 2017). Furthermore, in a 20-week randomized controlled trial with PTSD participants, alpha-rhythm EEG-NFB resulted in significantly reduced PTSD severity scores and promoted neuroplastic resynchronization (i.e., homeostatic rebound) of alpha power within the anterior DMN, a region which showed decreased alpha power at baseline (Nicholson et al., 2023). Taken together, the PCC may be uniquely situated as a neurofeedback target region that can generate wide-reaching homeostatic regulation of pathological brain dynamics. As previously discussed, emerging research has identified the PCC as critically implicated in PTSD, where increased PCC activation is observed during the reliving and reexperiencing of trauma-related memories (Ramage et al., 2013; Frewen et al., 2017; Awasthi et al., 2020; Thome et al., 2020) and decreased PCC activation is associated with longitudinal improvements in PTSD symptoms (Ke et al., 2016; Garrett et al., 2019). In conjunction with the unique neural mechanisms and symptom decreases that were observed in the current study for the PCC group, these network-level perspectives on PCC functionality suggest that it may be a particularly effective neurofeedback target region in the context of PTSD.

### 4.4 Limitations and future directions

Limitations of the present analysis include a small sample size which may have limited our ability to detect significant between-group differences in state changes in PTSD symptoms and may also impact the generalizability of the neural activation findings. Additionally, data for the two participant groups were collected sequentially which precluded randomized group assignment. Although broadly similar themes emerged for both groups regarding the use of regulatory strategies (i.e., mindfulness-based techniques, positive self-talk, visual imagery), future research is needed to comprehensively evaluate strategy use and its implications on training success. In order to further investigate the potential therapeutic effect of neurofeedback in PTSD, exploration of complementary neurofeedback modalities with optimized temporal resolution is warranted (i.e., EEG, MEG). Indeed, in a 20-week randomized controlled trial by our group, EEG-NFB targeting alpha oscillations (related to PCC activity) was shown to be associated with significant reductions in PTSD symptom severity (Nicholson et al., 2020b, 2023). Further research is needed to assess how the temporal and spatial resolutions of different neurofeedback modalities (i.e., fMRI, EEG, MEG) might impact regulation success and clinical outcomes. Lastly, it is important to note that the present study was not preregistered as data collection began before preregistration was a standard practice in the field. To address the limitations of the current study and further elucidate the effect of neurofeedback target selection in PTSD, our group has preregistered and is currently conducting a multisession, double-blind, randomized controlled trial comparing PCC and amygdala neurofeedback targets versus a sham-control arm (NCT05456958). As part of this study, we are also conducting semi-structured interviews with participants after each neurofeedback session in order to obtain qualitative data on their use of regulatory strategies. Including a sham control arm in the study will provide further insight into the neurophysiological specificity of both neurofeedback target regions. Examining the impact of multiple rt-fMRI-NFB sessions on brain activation and clinical outcomes will reveal the potential cumulative effects of rt-fMRI-NFB and inform the development of more effective treatment strategies.

## 5 Conclusion

In summary, we compared neural activation between two groups of PTSD participants who were trained to downregulate activity within one of two clinically relevant target regions – the amygdala or PCC – using rt-fMRI-NFB. Although both participant groups were able to downregulate activity within their respective target brain regions to a similar extent, we observed significant differences between the groups in terms of neural activity and clinical outcomes during neurofeedback-mediated regulation. Indeed, the PCC group as compared to the amygdala group showed widespread whole-brain decreases in activity within the bilateral cuneus/precuneus/primary visual cortex, the left superior parietal lobule, the left occipital pole, and the right superior temporal gyrus/TPJ. Conversely, for the amygdala group as compared to the PCC group, there were no significant unique (i.e., over and above that of the PCC group) decreases in neural activity. These differential neural results may help to explain the finding that only the PCC group showed improvements in state PTSD symptoms (i.e., reliving and distress) during neurofeedback training. Although altered activation within both the PCC and amygdala are widely reported among PTSD populations, emerging evidence from studies employing a network-level neuroscience perspective suggests that the PCC – due to its heterogeneous functionality, highly connected nature, and involvement in regulating both regional and global neural dynamics – may be a highly effective target for downregulation using rt-fMRI-NFB in PTSD.

## Data availability statement

The data that support the findings of this study are available from the corresponding author upon reasonable request. Requests to access these datasets should be directed to AN, andrew.nicholson@theroyal.ca.

## Ethics statement

The studies involving humans were approved by the Western University Health Sciences Research Ethics Board (HSREB) (project ID: 103933) and the Lawson Health Research Institute (project ID: 1975). The studies were conducted in accordance with the local legislation and institutional requirements. The participants provided their written informed consent to participate in this study.

## Author contributions

RL and AN conceived and designed the original research study. DR, MD, PF, DS, FS, JT, RN, RJ, TR, RL, and AN were involved in data collection and execution of the research study. JL and AN conceived of the research questions, conducted the statistical analyses and interpretation of the data, and drafted and revised the manuscript. BF and NH-K provided valuable input on statistical analyses and interpreting the results. All authors contributed to the editing and revision of the final manuscript for submission.

## Footnotes

^1^ http://www.rtfin.org/CREDnf

^2^ https://www.nitrc.org/projects/artifact_detect
