# Supplementary material for: A tale of two targets: examining the differential effects of posterior cingulate cortex- and amygdala-targeted fMRI-neurofeedback in a PTSD pilot study
Source: Front Neurosci. 2023 Nov 29;17:1229729. doi: 10.3389/fnins.2023.1229729 (PMC10716260; doi:10.3389/fnins.2023.1229729)
Supplement: Supplementary file 1 [file Data_Sheet_1.PDF]

CRED-nf checklist summary

03 October, 2023

Manuscript title: A Tale of Two Targets: Examining the Differential Effects of Posterior Cingulate Cortex- and Amygdala-targeted fMRI-Neurofeedback in a PTSD Pilot Study

Corresponding Author: Dr. Andrew A. Nicholson
Corresponding author email: andrew.nicholson@theroyal.ca

Item No.	Checklist item	Manuscript Details
**Pre-experiment**		
1a	Pre-register experimental protocol and planned analyses	*This experiment was not preregistered*
1b	Justify sample size	The sample size of this pilot investigation was based on study feasibility during the recruitment period.
**Control groups**		
2a	Employ control group(s) or control condition(s)	Our neurofeedback protocol consisted of three conditions: regulate, view, and neutral. In the regulate condition, participants were instructed to decrease the neurofeedback signal while viewing a personalized trauma-related word. In the view condition, participants viewed a trauma-related word but were instructed to respond naturally and not attempt to exert regulatory control over the neurofeedback signal. In the neutral condition, participants viewed a personalized neutral word and were instructed to respond naturally and not attempt to exert regulatory control over the neurofeedback signal.
2b	When leveraging experimental designs where a double-blind is possible, use a double-blind	*NA: A double-blind was not appropriate for this experiment*
2c	Blind those who rate the outcomes	*Those who rated the outcome were not blind to group assignment*
	Blind those who analyse the data	*Those who analysed the data were not blind to group assignment*
2d	Examine to what extent participants and experimenters remain blinded	*No measures were taken to examine whether participants and experimenters remained blind*
2e	In clinical efficacy studies, employ a standard-of-care intervention group as a benchmark for improvement	*NA: This is not a clinical efficacy study*
**Control measures**		

3a	Collect data on psychosocial factors	Prior to scanning, participants completed several clinical assessments, including the Beck's Depression Inventory (BDI) (Beck et al., 1997), the Childhood Trauma Questionnaire (CTQ) (Bernstein et al., 2003) and the Multiscale Dissociation Inventory (MDI) (Briere et al., 2005). After each of the fMRI neurofeedback runs, participants completed the Response to Script Driven Imagery Scale (RSDI) (Hopper, Frewen, Sack, et al., 2007), which included the following symptom subscales: reliving, distress, physical reactions, dissociation, and emotional numbing.
3b	Report whether participants were provided with a strategy	Participants were instructed that they would be regulating an area of the brain related to emotion. To avoid biasing the selection and usage of regulatory mental strategies by participants, no specific instructions were provided on how to regulate the neurofeedback target region (Nicholson et al., 2018, 2021; Nicholson, Rabellino, et al., 2016; Paret et al., 2014; Paret, Kluetsch, et al., 2016). Rather, participants were instructed to use whichever strategies they personally found to work best for regulating the neurofeedback signal.
3c	Report the strategies participants used	Participants in both groups reported using similar regulatory strategies including mindfulness-based techniques, positive self-talk, and the use of visual imagery.
3d	Report methods used for online-data processing and artifact correction	For all participants, we performed identical procedures to present real-time neural activation of the neurofeedback target region via a thermometer display. First, we imported anatomical scans into BrainVoyager (version QX2.4, Brain Innovations), skull-stripped and transformed them into Talairach space. We then added the normalization parameters into Turbo-BrainVoyager (TBV, version 3.0, Brain Innovations) which was the software used for real-time processing and analysis of BOLD signals. During real-time signal processing, TBV detected and corrected for small head movements (via rigid body transformation to the first recorded volume) and conducted spatial smoothing (4-mm full-width-half-maximum; (FWHM). We discarded the first two volumes of the functional scans before real-time processing.
3e	Report condition and group effects for artifacts	*Condition and group effects for artifacts were not measured, or not reported in the manuscript*
**Feedback specifications**		

4a	Report how the online-feature extraction was defined	Next, we defined the neurofeedback target region using TBV. For the amygdala, we used a bilateral anatomical mask from the PickAtlas software (WFU Pickatlas). For the PCC, we used a 6 mm sphere at the coordinate (MNI: 0 -50 20) (Bluhm et al., 2009). In both cases, we then used the "best voxel selection" tool in TBV to calculate the BOLD signal amplitude in the defined target area. This method identifies the 33% most active (i.e., the highest beta-values) voxels for the view > neutral contrast. The first two trials of each neurofeedback run were the view and neutral conditions, which allowed us to select voxels based on the view > neutral contrast. This selection was dynamically updated throughout the duration of training. Indeed, as outlined in previous publications (Nicholson et al., 2018; Nicholson, Rabellino, et al., 2016; Paret et al., 2014; Paret, Ruf, et al., 2016), dynamic voxel selection is based on (a) the voxel with the largest beta value, and (b) the magnitude of deviation from the mean of all condition betas (Goebel, 2014). This method ensures that there are no inter-subject differences in the number of voxels used for signal extraction. Additionally, it accounts for slight shifts in anatomical delineation resulting from changes in alignment across runs and/or movement-related slice shifts. The ne
		urofeedback signal was calculated as the mean of the processed BOLD signal over the included voxels within the target brain region. In order to smooth out rapid BOLD signal fluctuations, the neurofeedback signal shown to participants via thermometer display was the mean of the neurofeedback signal of the current and 3 preceding TRs (Nicholson et al., 2021; Nicholson, Rabellino, et al., 2016; Paret et al., 2014; Paret, Ruf, et al., 2016). At the start of each trial, the mean of the neurofeedback signal of the first 4 TRs (preceding stimuli onset) were utilized as a baseline and shown to participants as an orange line on the thermometer display. Subsequently, the level of the thermometer was continuously updated (at each TR) and shown to participants throughout the 3 neurofeedback training runs. Each segment of the thermometer corresponded to a 0.2% change in BOLD activation, with a maximum range of +2.8% and -1.2% from baseline (Nicholson et al., 2018, 2021; Nicholson, Rabellino, et al., 2016; Paret et al., 2014; Paret, Ruf, et al., 2016).
4b	Report and justify the reinforcement schedule	*The manuscript does not report or justify the reinforcement schedule*

4c	Report the feedback modality and content	All participants underwent an identical experimental protocol and neurofeedback paradigm, with the exception of the neurofeedback target region (i.e., the amygdala or PCC) (Figure 1). During neurofeedback training, participants were presented with a signal corresponding to BOLD activation within the neurofeedback target region. This neurofeedback signal was presented as a virtual thermometer on both sides of the MRI screen that was visible to participants while they were inside the scanner. The bars on the thermometer increased or decreased in correspondence to changes in BOLD activation within the neurofeedback target region. Participants were instructed that they would be regulating an area of the brain related to emotion. To avoid biasing the selection and usage of regulatory mental strategies by participants, no specific instructions were provided on how to regulate the neurofeedback target region (Nicholson, Rabellino, et al., 2016; Nicholson et al., 2018, 2021; Paret et al., 2014; Paret, Kluetsch, et al., 2016). Rather, participants were instructed to use whichever strategies they personally found to work best for regulating the neurofeedback signal. Participants were also asked to focus their gaze directly on the presented word for the duration of each condition and to use their peripheral vision to mo
		nitor the thermometers. Participants were also informed that the neurofeedback signal lags behind their brain activity by approximately 6-8 seconds (due to the BOLD signal time lag).
4d	Collect and report all brain activity variable(s) and/or contrasts used for feedback, as displayed to experimental participants	Next, we defined the neurofeedback target region using TBV. For the amygdala, we used a bilateral anatomical mask from the PickAtlas software (WFU Pickatlas). For the PCC, we used a 6 mm sphere at the coordinate (MNI: 0 -50 20) (Bluhm et al., 2009). In both cases, we then used the "best voxel selection" tool in TBV to calculate the BOLD signal amplitude in the defined target area. This method identifies the 33% most active (i.e., the highest beta-values) voxels for the view > neutral contrast. The first two trials of each neurofeedback run were the view and neutral conditions, which allowed us to select voxels based on the view > neutral contrast. This selection was dynamically updated throughout the duration of training. Indeed, as outlined in previous publications (Nicholson et al., 2018; Nicholson, Rabellino, et al., 2016; Paret et al., 2014; Paret, Ruf, et al., 2016), dynamic voxel selection is based on (a) the voxel with the largest beta value, and (b) the magnitude of deviation from the mean of all condition betas (Goebel, 2014). This method ensures that there are no inter-subject differences in the number of voxels used for signal extraction. Additionally, it accounts for slight shifts in anatomical delineation resulting from changes in alignment across runs and/or movement-related slice shifts. The ne

		urofeedback signal was calculated as the mean of the processed BOLD signal over the included voxels within the target brain region. In order to smooth out rapid BOLD signal fluctuations, the neurofeedback signal shown to participants via thermometer display was the mean of the neurofeedback signal of the current and 3 preceding TRs (Nicholson et al., 2021; Nicholson, Rabellino, et al., 2016; Paret et al., 2014; Paret, Ruf, et al., 2016). At the start of each trial, the mean of the neurofeedback signal of the first 4 TRs (preceding stimuli onset) were utilized as a baseline and shown to participants as an orange line on the thermometer display. Subsequently, the level of the thermometer was continuously updated (at each TR) and shown to participants throughout the 3 neurofeedback training runs. Each segment of the thermometer corresponded to a 0.2% change in BOLD activation, with a maximum range of +2.8% and -1.2% from baseline (Nicholson et al., 2018, 2021; Nicholson, Rabellino, et al., 2016; Paret et al., 2014; Paret, Ruf, et al., 2016).
4e	Report the hardware and software used	We used the same 3 Tesla MRI Scanner (Siemens Biograph mMR) at the Lawson Health Research Institute for all participants. Stimuli were presented using Presentation software from Neurobehavioral Systems. For all participants, we performed identical procedures to present real-time neural activation of the neurofeedback target region via a thermometer display. First, we imported anatomical scans into BrainVoyager (version QX2.4, Brain Innovations), skull-stripped and transformed them into Talairach space. We then added the normalization parameters into Turbo-BrainVoyager (TBV, version 3.0, Brain Innovations) which was the software used for real-time processing and analysis of BOLD signals. During real-time signal processing, TBV detected and corrected for small head movements (via rigid body transformation to the first recorded volume) and conducted spatial smoothing (4-mm full-width-half-maximum; (FWHM).
5a	Report neurofeedback regulation success based on the feedback signal	Previously, we found that individuals with PTSD were able to significantly downregulate BOLD activity within the PCC (Nicholson et al., 2021) and amygdala (Nicholson, Rabellino, et al., 2016) during regulate as compared to view conditions for all three neurofeedback training runs, as well as the transfer run. Follow-up independent sample t-tests revealed that there was no significant difference in the average event-related BOLD response within the target region between the two groups during regulate or view in any individual neurofeedback run (regulate, run 1: $t(26) = .197$, $p = .846$, Cohen's $d = .074$; regulate, run 2: $t(26) = .342$, $p = .735$, Cohen's $d = .129$; regulate, run 3: $t(26) = .637$, $p = .530$, Cohen's $d = .241$; regulate, run 4: $t(26) = .794$, $p = .435$, Cohen's $d = .300$; view, run 1: $t(26) = -1.11$, $p = .278$, Cohen's $d = -.419$; view, run 2: $t(26) = -.064$, $p = .949$, Cohen's $d = -.024$; view, run 3: $t(26) = .045$, $p = .965$, Cohen's $d = .017$; view, run 4: $t(26) = -.645$, $p = .525$, Cohen's $d = -.244$) (Figure 2).

5b	Plot within-session and between-session regulation blocks of feedback variable(s), as well as pre-to-post resting baselines or contrasts	*The manuscript does not plot within-session and between-session regulation blocks of feedback variable(s), as well as pre-to-post resting baselines or contrasts*
5c	Statistically compare the experimental condition/group to the control condition(s)/group(s) (not only each group to baseline measures)	*The manuscript does not statistically compare the experimental condition/group to the control condition(s)/group(s)*
**Outcome measures - behaviour**		
6a	Include measures of clinical or behavioural significance, defined a priori, and describe whether they were reached	In summary, when assessing state changes in PTSD symptoms, we observed clear differences in terms of within-group results for the PCC and amygdala groups. As previously published, PCC downregulation was found to show a significant main effect of run for the nonparametric ANOVA investigating reliving (2 (3) = 11.49, p = .009) and distress (2 (3) = 13.79, p = .003) symptoms, and non-significant effects for the other RSDI subscales [physical reactions: 2 (3) = 4.70, p = .195; emotional numbing: 2 (3) = 2.26, p = .520; dissociation: 2 (3) = 2.29, p = .515] when controlling for multiple comparisons (Nicholson et al., 2021). Wilcoxon signed-rank tests revealed lower reliving scores during run 3 versus run 1 (p = .016) and lower distress scores during runs 3 (p = .010) and 4 (p = .013) versus run 1 for the PCC group (Nicholson et al., 2021) (Table 5). By contrast, amygdala downregulation was not found to show a significant main effect of run for any of the nonparametric ANOVAs that were conducted for each of the RSDI subscales [reliving: 2 (3) = 9.21, p = .027; distress: 2 (3) = 4.98, p = .173; physical reactions: 2 (3) = 10.24, p = .017; emotional numbing: 2 (3) = .240, p = .971; dissociation: 2 (3) = .241, p = .971] when controlling for multiple comparisons. Wilcoxon signed-rank tests did not reveal any
		significant differences in RSDI scores between runs (i.e., run 1 vs run 3; run 1 vs run 4) for the amygdala group. When directly comparing the two groups at during each timepoint run using Mann-Whitney U tests, there were no significant differences observed for any of the RSDI subscale scores (Table 6).
6b	Run correlational analyses between regulation success and behavioural outcomes	*This manuscript does not compare regulation success and behavioural outcomes*
**Data storage**		
7a	Upload all materials, analysis scripts, code, and raw data used for analyses, as well as final values, to an open access data repository, when feasible	*No additional documents related to the materials, analysis scripts, code, raw data, or final values are available for this manuscript*
